# The response to influenza vaccination is associated with DNA methylation-driven regulation of T cell innate antiviral pathways

**DOI:** 10.21203/rs.3.rs-4324518/v1

**Published:** 2024-05-23

**Authors:** Hongxiang Fu, Harry Pickering, Liudmilla Rubbi, Ted M. Ross, Wanding Zhou, Elaine F. Reed, Matteo Pellegrini

**Affiliations:** University of California Los Angeles; University of California Los Angeles; University of California Los Angeles; University of Georgia; The Children’s Hospital of Philadelphia; University of California Los Angeles; University of California Los Angeles

**Keywords:** DNA methylation, influenza virus, influenza vaccine, targeted bisulfite sequencing, RNA sequencing, cell-type deconvolution

## Abstract

**Background:**

The effect of vaccination on the epigenome remains poorly characterized. In previous research, we identified an association between seroprotection against influenza and DNA methylation at sites associated with the RIG-1 signaling pathway, which recognizes viral double-stranded RNA and leads to a type I interferon response. However, these studies did not fully account for confounding factors including age, gender, and BMI, along with changes in cell type composition.

**Results:**

Here, we studied the influenza vaccine response in a longitudinal cohort vaccinated over two consecutive years (2019–2020 and 2020–2021), using peripheral blood mononuclear cells and a targeted DNA methylation approach. To address the effects of multiple factors on the epigenome, we designed a multivariate multiple regression model that included seroprotection levels as quantified by the hemagglutination-inhibition (HAI) assay test.

**Conclusions:**

Our findings indicate that 179 methylation sites can be combined as potential signatures to predict seroprotection. These sites were not only enriched for genes involved in the regulation of the RIG-I signaling pathway, as found previously, but also enriched for other genes associated with innate immunity to viruses and the transcription factor binding sites of BRD4, which is known to impact T cell memory. We propose a model to suggest that the RIG-I pathway and BRD4 could potentially be modulated to improve immunization strategies.

## Background

Influenza vaccination has been recognized as the most effective method to prevent influenza virus infections and their complications, thereby reducing morbidity and mortality (1). The development and deployment of influenza vaccines are guided by ongoing surveillance of circulating influenza strains, as the virus exhibits significant antigenic variability. This variability necessitates the annual reformulation of the influenza vaccine to match the predicted predominant circulating strains, highlighting the challenges in vaccine effectiveness and the need for continuous evaluation. Influenza vaccines work by triggering the production of antibodies against the hemagglutinin surface glycoprotein (2). These vaccines are designed to elicit hemagglutinin-specific antibody levels in the body, which can be quantified through the hemagglutination inhibition (HAI) assay. This assay measures the presence of functional antibodies capable of preventing the agglutination of turkey red blood cells by the virus.

Numerous studies have sought to link the response to the influenza vaccine with various factors, including age, body mass index (BMI), and gene expression (3). Despite these efforts, research into the epigenetic mechanisms influencing vaccine responses remains incomplete. Associating epigenetic signatures directly with vaccine responsiveness is challenging due to the influence of confounding factors, such as age and BMI, which also impact the epigenome. In this study, we focus on DNA methylation (DNAm), which is a key epigenetic mechanism that plays a crucial role in gene regulation, impacting a wide array of biological functions and disease processes (4–6). It involves the addition of a methyl group to the fifth carbon position on cytosine residues. In the human genome, DNAm primarily occurs within the context of CpG dinucleotides. The distribution of methylation across the genome is uneven, with CpG islands typically exhibiting low levels of methylation (7). Advances in next-generation sequencing (NGS) technologies have enabled the detailed profiling of DNAm patterns by analyzing bisulfite-treated DNA. Techniques such as whole-genome bisulfite sequencing (WGBS) and targeted bisulfite sequencing (TBS) offer precise measurements of DNAm at CpG sites (8).

In this study, we chose to profile our samples using TBS because it offers compelling advantages for the detailed analysis of DNAm patterns, especially in specific regions of interest within the genome. By focusing on predefined genomic regions, TBS enables researchers to achieve high-resolution, accurate measurements of methylation levels at CpG sites within specific target regions (9).

Previously, we identified longitudinal changes of DNAm and found these changes were significant in genes associated with viral response pathways, such as the regulation of RIG-1 signaling, which leads to the production of interferons (10). Complementary to our findings, another investigation utilized mice with site-specific genetic modifications and tools for targeted demethylation to establish a direct link between methylation patterns and the production of type I interferons (11). Furthermore, an additional study pinpointed methylation sites that were associated with the humoral response to influenza vaccination. This research revealed that methylation sites predictive of humoral immune responses were notably enriched for the binding sites of polycomb-group proteins and the FOXP2 transcription factor, underscoring the intricate relationship between epigenetic modifications and immune system function (12).

However, these studies didn’t address important confounders that may affect DNAm and influenza vaccination response. Notably, DNAm changes are associated with aging (13), and its patterns are highly specific to different cell types (14). To address these potential confounding factors, including age, BMI, and cell type proportions, our study adopted a multifactorial approach. We included the same cohort consecutively vaccinated over two years and conducted RNA sequencing (RNA-seq) for year 2020. This comprehensive approach led to the identification of key methylation sites associated not only with the previously identified RIG-I signaling pathway but also with other genes involved in innate immunity to viruses (10). Our findings illuminate the complex interaction between DNAm and the influenza vaccine response, accounting for various potential confounders. The genes we identified may offer novel insights for future vaccine development.

## Results

### Study cohort and serological response to influenza vaccination

**Figure 1:**
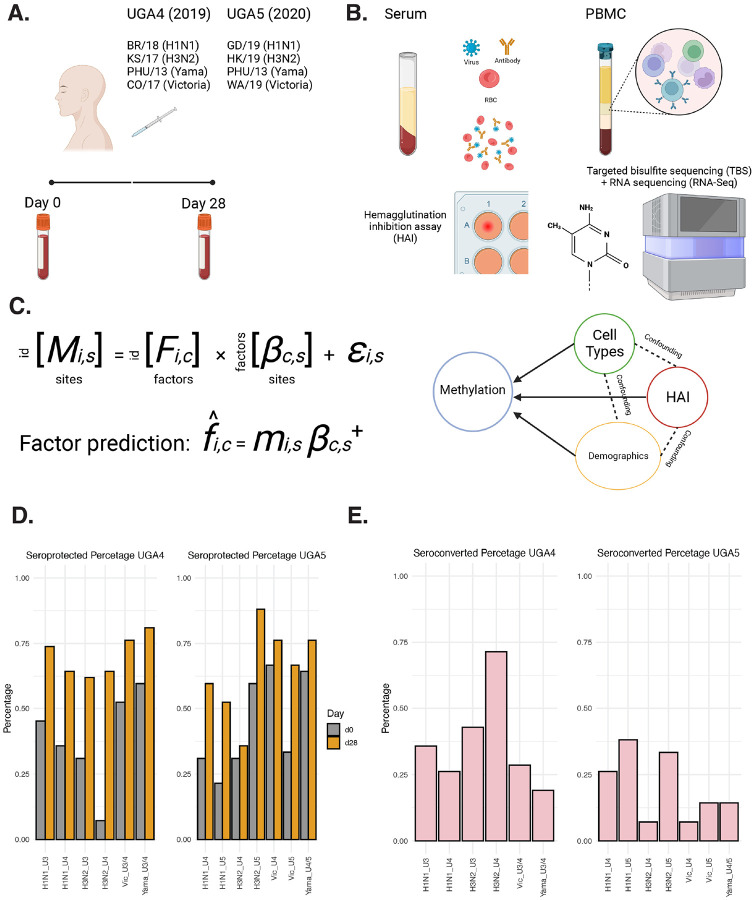
Study schematic and design. (A). Vaccination summary of UGA4 (2019) and UGA5 (2020), the composition for quadrivalent Fluzone influenza vaccine in each cohort. (B) Hemagglutination inhibition assay, targeted bisulfite sequencing, and RNA sequencing schematic. (C) Model illustration, multivariate multiple regression and pseudoinverse were used to address the effects of multiple factors on the epigenome. (D) The proportion of seroprotected subjects at day 0 and 28 days after receiving the vaccination. (E) The proportion of seroconverted subjects 28 days after receiving the vaccination.

For our study, we focused on the cohorts comprising influenza vaccine recipients, enrolled at the University of Georgia between 2016 and 2020 (15). From these cohorts, we selected a subset of 42 individuals who participated in the 2020 vaccination program (UGA5) and had also received their influenza vaccinations during the preceding 2019 program (UGA4). The administered vaccine was Fluzone (Fig. 1A), an inactivated, quadrivalent construct consisting of one strain from each of the four major subtypes (H1N1, H3N2, Yamagata, and Victoria) as described in detail in the Materials and Methods section.

Hemagglutination inhibition assay was used to quantify the amount of antibody to influenza viruses. For both cohorts, peripheral blood mononuclear cells were collected. Both targeted bisulfite sequencing and RNA sequencing were performed at baseline prior to vaccination and 28 days following vaccination (Fig. 1B). We sequenced approximately 5500 targeted regions within the genome that were selected based on different criteria as described in detail the Methods section (Additional le 2: Supplementary Table 1). These sites were selected to be potentially involved in aging, viral responses, and cellular metabolisms. In total, we quantified levels of DNAm at approximately 100,000 CpG sites. The final methylation matrix contained 22,740 CpG sites that were covered by at least 20 reads in all the samples as described in detail in the Methods section. The multivariate model was then utilized to analyze the methylation levels with immune response under various cofounding variables (Fig. 1C).

Seroprotection is defined as HAI antibody titers > = 1:40 or more post vaccination, and seroconversion is defined as a fourfold increment in titer compared with the baseline, leading to a titer of 1:40 or more. Each strain within the cohorts had a seroprotected percentage exceeding 50% at 28 days post-vaccination for both UGA4 and UGA5. In particular, more than 80% of the subjects were seroprotected against the H3N2 strain in UGA5 (Fig. 1D). H3N2 in UGA4 was the strain with the highest seroconverted rate, nearly 75% of the subjects seroconverted 28 days after vaccination. Overall, UGA4 participants had a higher percentage of seroconvertion compared to participants in UGA5 (Fig. 1E).

### Cell type deconvolution across four time points were correlated with different serology quantifications

**Figure 2:**
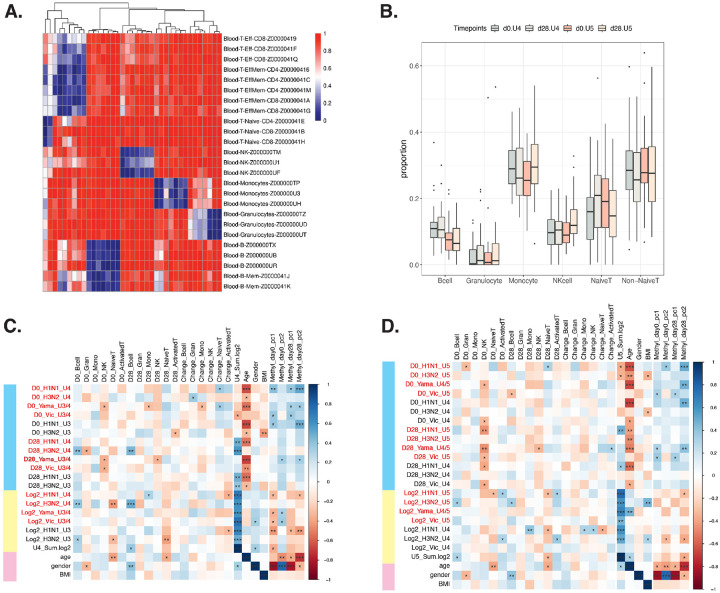
Analysis of cell type proportion estimation in two cohorts and their association with phenotypic traits. (A) 37 differentially methylated regions of the recently published methylation atlas, blue indicates hypomethylation and red indicates hypermethylation. (B) Results of cell type deconvolution for cohorts UGA4 and UGA5, assessed at two different time points. 6 cell types were selected which occur in PBMC. (C) Correlation of UGA4 cell type estimates with various phenotypic traits. Blue rows are HAI measurements (U3/4 indicates the vaccine strain is the same in UAG3 and UGA4), yellow rows are log2 fold change of HAI from day 0 to day 28 (sum log2 is the sum of log2 fold change of the vaccine strain used in U4), and pink rows are other factors. (D) Correlation of UGA5 cell type estimates with various phenotypic traits (*Indicates p < 0.05, **Indicates p < 0.01, *** Indicates p < 0.001).

Methylation is known to be cell type specific. To address the confounding factor of relative immune cell types in each PBMC sample, we conducted cell type deconvolution analysis using CellFi (16) from the published methylation atlas of various human cell types (17). We focused on cell types present in PBMCs and identified 37 differentially methylated regions (DMRs) for downstream cell type abundance estimation (Fig. 2A). The relative abundance of 6 immune cell types was estimated across two cohorts at both time points (Fig. 2B). Our results align with the known composition of PBMC cells (18), with ~ 70% being lymphocytes (including B cells, T cells, and Natural Killer (NK) cells), 25% monocytes, and a small percentage of granulocytes. To further validate our results, we utilized RNA sequencing data for the UGA5 cohort for estimating cell type abundances. The cell type proportions in UGA5 showed consistency between methylation and transcriptomic analyses (Additional file 1: Figure S6).

We analyzed the association between cell types and principal components of methylation with seroprotection levels (Figs. 2C–D). We observed various significant associations between cell type estimates and serology trait measurements. These correlations matched previous findings. For example, day 0 NK cells showed negative associations with HAI titers for multiple vaccine strains, which is consistent with their role in inhibiting adaptive immune responses and impacting immune memory post-vaccination (19, 20). Additionally, age was negatively associated with HAI titers (Additional file 1: Figure S1), emphasizing its known impact on vaccine response. We also found correlations between the principal components of DNA methylation level matrices and immune/biological traits. For example, the first three PCs showed strong gender association, and the fourth PC showed strong age association. They also demonstrated several significant positive correlations with immune responses to various strains, such as H1N1. This further reveals the importance of using a multivariate model to fully uncover the potential DNA methylation role in vaccination immune response.

### Cross-validation of the multivariate multiple regression model showed nearly accurate predictions of all the variables

**Figure 3:**
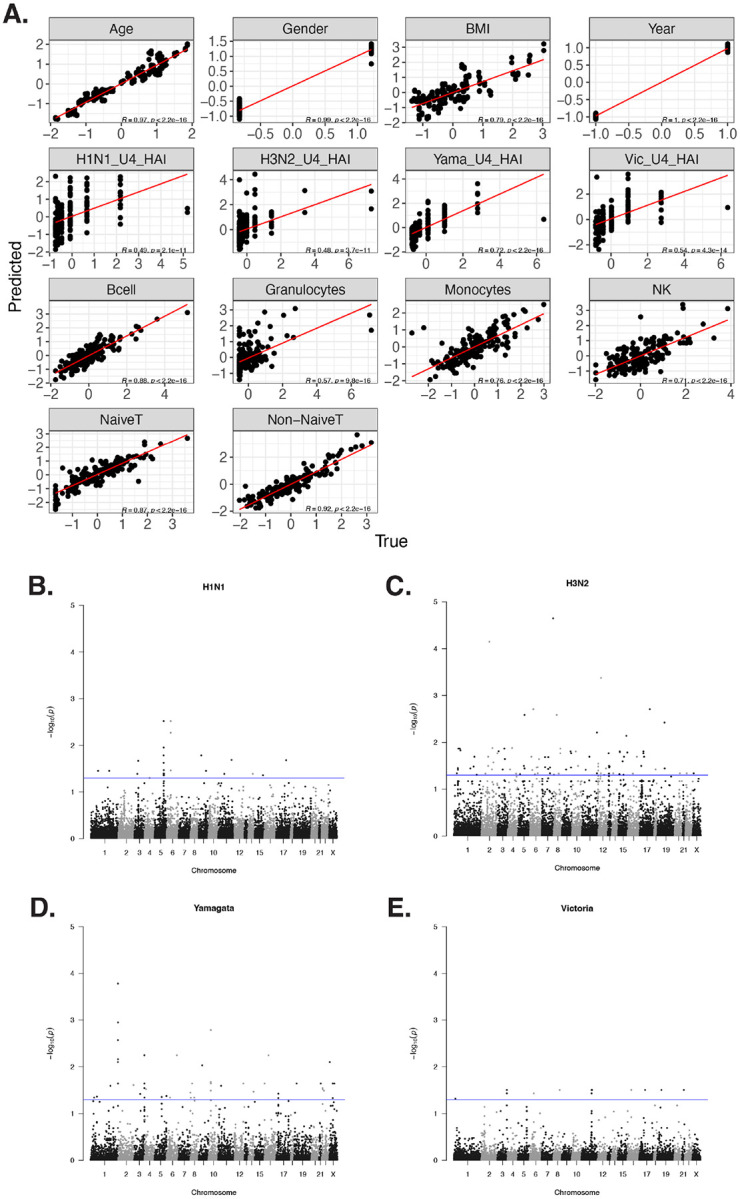
Multivariate multiple regression model LOOCV assessment and HAI significant coefficients. (A) LOOCV of the prediction from the multivariate multiple regression and pseudoinverse. The Pearson correlation between true and predicted of all the traits are all significant. (B) Manhattan plot of the significant coefficients of all the CpG sites for the four vaccine strains. Each dot represents the −log10 of FDR adjusted p values for the coefficients, and the blue line indicates the significant threshold.

We utilized a multivariate multiple regression model framework to model the relationship between DNA methylation levels as the response and vaccine serological immune levels and various confounding variables as features. We calculated the Pearson correlation between these variables and found that the absolute values of the correlations between pairs of variables were all below 0.5 (Additional file 1: Figure S2A). This indicates a lack of multicollinearity among them. The details of the model can be found in the methods section. To boost statistical power, we combined the two cohorts together and incorporated a year variable to mitigate the potential batch effect. We then applied leave-one-out cross-validation (LOOCV) to assess the model performance and prediction accuracy (Fig. 3A). This process revealed that our model yields accurate predictions of age, corroborating existing research that methylation can be utilized to construct aging clocks (21). Moreover, all the other variables also exhibited high Pearson correlation coefficients between predicted and true values, and all of them are statistically significant below the threshold of 0.05. To interpret the model, we examined the coefficient of the regression for each CpG site and each factor. Four Manhattan plots all showed significant sites from t-test for the four vaccine strains used here that participants received in 2019 (Fig. 3B). The full coefficient p value heatmap is shown in Figure S2B. Sites with significant gender-specific methylations were all located on the X chromosome.

### Significant HAI sites were enriched in virus response pathway and transcription factors binding sites

We performed enrichment analyses on the combined significant CpG sites identified as associated with HAI titer against each vaccine strain. To differentiate between the effects of hypermethylation and hypomethylation, we analyzed the model’s coefficients separately. Positive coefficients suggest that an increase in methylation levels is associated with higher HAI. While hypermethylation of gene promoter regions generally correlates with repression of gene expression, methylation in gene bodies can be associated with active gene expression (22). Conversely, hypomethylation often correlates with increased gene expression. 147 positive coefficients of HAI significant CpG sites were first mapped to the proximal genes, and gene set enrichment results showed viral response related pathways against the full probe background to avoid selection bias. Notably, genes like C1QBP and RNF125, identified in this process, have been previously implicated in the RIG-I signaling pathway, as observed in the longitudinal study of the UGA4 cohort (10). Type 1 interferon production is induced downstream of the RIG-I signaling pathway, and negative regulation of type 1 interferon production was significantly enriched for in hypermethylated CpG sites (Table 1).

**Figure 4:**
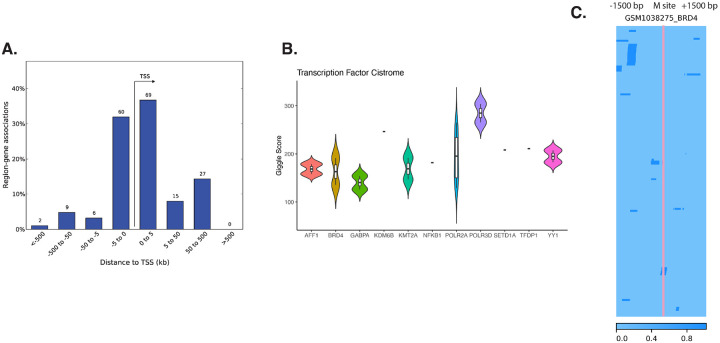
Transcription factors binding sites enrichment of significant positive HAI coefficients. (A) Distance from significant positive HAI coefficient sites to transcription start sites percentage. (B) Significant transcription factor binding sites from the Cistrome database, specifically focusing on either blood or immune related sources. (C) Overlap between methylation site and publicly available ChIP-Seq data. Dark blue color marks the BRD4 transcription factor binding sites.

We extended our analysis to identify transcription factor binding sites enriched at our significant CpGs using the Cistrome database (Fig. 4B), and we filtered the sources of the ChIP data to be blood or immune related. Among the enriched transcription factors, Brd4 and NFKB1 are known to regulate immune responses (23, 24). We also cross-referenced our findings with publicly available Brd4 ChIP-Seq data (7), revealing the binding sites that are proximal to the methylation site regions (Fig. 4C). Brd4 was previously found to influence T cell memory and mediate RIG-I upregulation. It is also required by interferon regulatory factors in respiratory syncytial virus infection (25).

The significant HAI-positive coefficients sites also showed a predominant enrichment in H3K27ac, an active enhancer mark known for its association with active transcription. This finding was further supported by chromatin accessibility data, suggesting that most positive sites are located in regions of open chromatin, indicative of active transcriptional activity (Additional file 1: Figure S4). Such a pattern was less apparent in HAI-negative sites. Our analysis also extended to examining the effects of age and Body Mass Index (BMI) on DNAm. We identified two transcription factors, Rest and Tal1, that significantly correlated with these traits, respectively. Rest plays a crucial role in preventing senescence phenotypes, while Tal1 is implicated in high risk of obesity (26, 27). These findings highlight Rest and Tal1 as potential targets for future studies focusing on immunosenescence and obesity in influenza vaccine response (Additional file 1: Figure S5). Significant HAI-negative sites notably overlapped with LMO2 (Additional file 1: Figure S3), a transcription factor previously identified as a key factor in the influenza virus response (28).

### Differential gene expression analysis of UGA5 complemented the methylation analysis result

**Figure 5:**
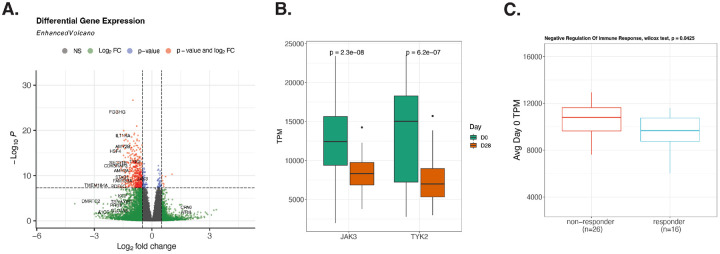
RNA gene expression analysis of UGA5 cohort. (A) Differential gene expression analysis between day 0 and 28 days after vaccine injection, (B) TPM of JAK3 and TYK2 between day 0 and 28 days after vaccine injections. (C) Average Day 0 TPM of Negative Regulation of Immune Response genes (PCBP2, TNFAIP3, FURIN, ATG12, ATG5) from significant HAI positive coefficients enrichment between responder and non-responder.

For the UGA5 cohort, RNA sequencing (RNA-seq) data were analyzed both before and after vaccination for the same patients. Initially, we performed a differential gene expression analysis using the raw transcript count data. This approach identified 574 genes that were differentially expressed, meeting the genome-wide significance threshold (Fig. 5A). Notably, two genes, JAK3 and TYK2, exhibited a significant decrease in transcription 28 days post-vaccination (Fig. 5B). These genes are known to act as signal transducers in the adaptive immune STAT pathways, which are downstream of interferon lambda signals originating from the innate immune RIG-I pathway (29). Interestingly, the genes corresponding to HAI-positive sites we found above did not show differential expression post-vaccination. However, we did observe a significant difference in the average Transcripts Per Million (TPM) for the enriched Negative Regulation of Immune Response genes from HAI-positive sites on day 0 between vaccine responders and non-responders (Fig. 5C), based on change in HAI titers from pre- to 28-days post-vaccination, which suggests a potential link between baseline gene expression levels under methylation modulation and vaccine response.

## Discussion

We developed a multimodal approach to explore the joint influence of multiple factors on DNA methylation and the humoral immune response to influenza vaccination. In this study, we focused on 42 subjects vaccinated with quadrivalent Fluzone across two consecutive seasons (UGA4 in 2019, UGA5 in 2020), noting that the vaccine composition differed between the two years except for the Yamagata strain. Notably, the UGA5 cohort exhibited a lower seroconversion rate compared to UGA4, potentially influenced by the concurrent COVID-19 pandemic (30, 31). For our model, we focused on the four vaccine strains from the UGA4 cohort, for which HAI titers were also quantified in UGA5.

Methylation is a cell type-specific epigenetic mechanism (17). Our cell type deconvolution provides an estimation of the proportion of PBMC cell types that can be included in the model. Interestingly, the proportion of NK cells was negatively correlated with vaccine immune response. By contrast, B cells and T cells were positively correlated with HAI seroresponse. NK cells play an important role in antiviral responses by expressing activation receptors in the innate immune system, whereas B cells and T cells function in the adaptive immune system producing antibodies and killing infected cells (32, 33).

When identifying methylation sites associated with serological immune responses to influenza, we also considered other factors, such as age and BMI, known to be associated with methylation changes. Our final multivariate multiple regression model demonstrated significant accuracy in predicting age, sex, cell types and HAI levels against all four strains, indicating the importance of these factors in mediating the methylation at the measured sites. One hundred and forty seven hypermethylated CpG sites were associated with HAI levels against four vaccine strains, suggesting gene expression inhibition of proximal genes. These sites were mostly mapped to Negative Regulation to Defense Response to Virus and Type 1 Interferons including genes involved in the negative regulation of the RIG-I signaling pathway. Hypermethylation of those sites leads to the inhibited expression of these negative regulators and therefore likely upregulation of type 1 interferon production.

Furthermore, we investigated the overlap of these significant methylation sites with transcription factor binding sites, which identified BRD4, a well-studied member of the bromodomain and extra-terminal protein family in immune diseases and cancer (34). Overlaps found in public ChIP-Seq data from blood or immune cells suggest a potential role for BRD4 in regulating the vaccine response. Additionally, differential gene expression analysis of the UGA5 cohort highlighted JAK3 and TYK2 as significantly differentially expressed following vaccination, underscoring the potential involvement of the JAK signaling pathway in this network.

**Figure 6:**
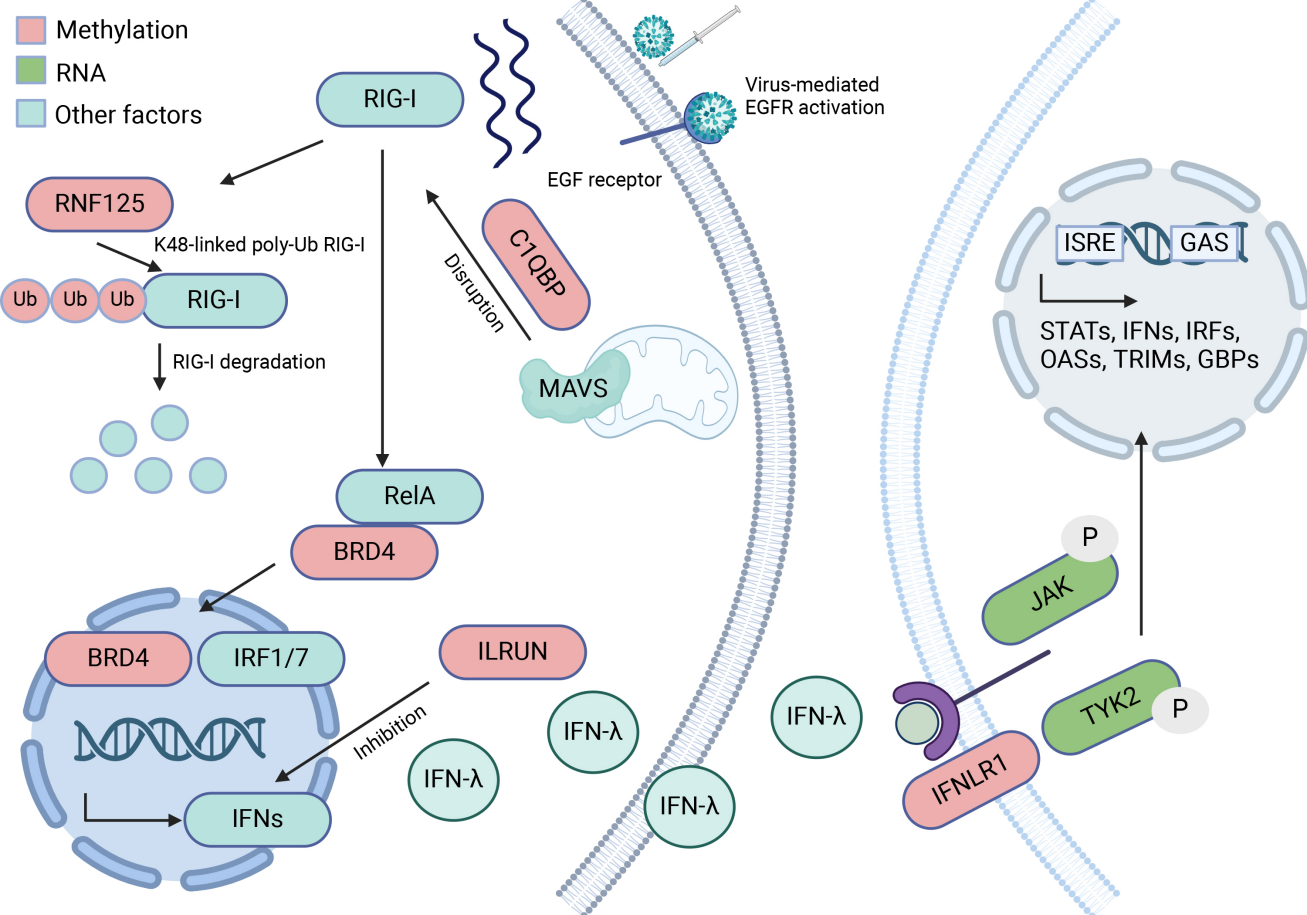
Generated using Biorender (Additional file 3), the full proposed model of multimodal DNA methylation analysis and differential gene expression analysis of immune responses to influenza vaccine (T cell B cell communication).

By combining the methylation multimodal model and differential gene expression results, we generated a final vaccine response model (Fig. 6). The methylation component (in red) mainly negatively regulates the RIG-I signaling pathway. After detecting the viral component, RIG-I signaling pathway is activated to drive the transcription of interferon production. The RIG-I signaling pathway was previously shown to be involved in antiviral responses and differential methylation analysis after influenza vaccination (10, 35, 36). Two genes whose methylation is significantly associated with HAI act as negative regulators of the RIG-I signaling pathway. C1QBP and its receptors are targeted to the mitochondrial outer membrane, and the interaction with MAVS led to the disruption of RIG-I signaling pathway (37). RNF125 interacts with RIG-I through proteasomal degradation after its conjugation to ubiquitin (38). The transcription factor BRD4, coupled with RelA, mediates RIG-I upregulation, which enhances interferon response factor IRF1/7 expression by transcriptional elongation (23, 25). The downstream output of interferons is regulated by ILRUN. ILRUN has UBA-like and NBR1-like domains which are essential for the inhibition of interferons (39).

The interferons produced by this signaling pathway can interact with the interferon lambda receptor 1 leading to signaling transduction. Interferon lambdas are innate immune cytokines that induce antiviral cellular responses (40). The differentially expressed genes we identified are associated with the JAK/TYK2 signaling pathway which activates downstream STAT proteins. A previous study has shown that The JAK family, including Tyk2, consists of tyrosine kinases linked to receptors that serve as signal transducers (29). The activation process of the JAK pathway begins when a cytokine, which can be interferon lambda, binds to IFNLR1. This binding induces a structural change in the receptor, which in turn activates and leads to the binding of JAK and Tyk2. These molecules form JAK dimers, and then phosphorylate the receptor, facilitating the binding, phosphorylation, and subsequent pairing of STAT proteins (STAT1, STAT2, STAT3, STAT4, STAT5a, STAT5b, and STAT6) which are important for both innate and adaptive immune response (29).

We postulate that the association between DNAm patterns and the response to influenza vaccination is primarily mediated through the activation of antiviral pathways in T cells. This hypothesis was corroborated by analyzing publicly available gene expression data for human immune cells from the Human Protein Atlas (41). Our analysis revealed that genes associated with HAI significant sites, namely RNF125, C1QBP, ILRUN, and BRD4, exhibit high levels of expression in T cells (Additional file 1:Figure S7). Moreover, the expression of the interferon lambda receptor, predominantly observed in naive B cells, suggests a critical role for T cell to B cell communication in eliciting an immune response to the vaccine. These findings underscore the importance of T cell-mediated pathways in the context of DNAm and its impact on vaccine-induced immunity.

Overall, our findings suggest potential targets for enhancing influenza vaccine efficacy through epigenetic and transcriptomic regulation of RIG-I and related immune pathways. Future research directions include single-cell methylation analysis to dissect cell-type-specific methylation patterns and their implications for vaccine response. For example, some of the genes and transcription factors might only appear in specific cell types, which would provide a clearer picture of the cell type specific methylation association with vaccine response. Additionally, expanding the study to include more subjects could enable strain-specific analyses. These studies will provide more insights into the influenza vaccine response through the scope of epigenetics.

## Conclusion

In conclusion, our study generated a multivariate multiple regression model to control for potential confounding variables when analyzing DNAm levels and their association with the influenza vaccine serological response. We identified significant methylation sites that are associated with the negative regulation of interferon production, along with the transcription factor BRD4, which plays a role in this regulatory process. Additionally, our differential RNA analysis shed light on the involvement of the JAK family genes in the signal transduction pathways of interferons. These insights not only enhance our understanding of the epigenetic and transcriptomic dynamics influencing vaccine efficacy but also highlight potential targets for future influenza vaccine design. By identifying specific genes and pathways that could be modulated to improve vaccine responses, this model framework sets the stage for more targeted and effective influenza vaccination strategies.

## Materials and Methods

### Vaccination and cohort subjects

As part of a longitudinal study led by the University of Georgia, Athens (UGA), a cohort of 690 individuals was recruited for five successive seasons from 2016 to 2020 (UGA 1–5) between the ages of 18 to 65 years old. Participants were administered the split-inactivated influenza vaccine, Fluzone, manufactured by Sanofi Pasteur. During the 2019–2020 season (UGA4), the vaccine strains incorporated were A/Brisbane/2018 (H1N1), A/Kansas/2017 (H3N2), B/Phuket/2013 (Yamagata lineage), and B/Colorado/2017 (Victoria lineage). In the following 2020–2021 season (UGA5), the strains included were A/Guangdong/2019 (H1N1), A/Hong Kong/2019 (H3N2), B/Phuket/2013 (Yamagata lineage), and B/Washington/2019 (Victoria lineage). The Institutional Review Board of the University of Georgia granted approval for the study protocols, informed consent procedures, and data gathering methodologies (IRB #3773). For this study, a subset comprising 42 participants, all of whom were enrolled in UGA 5 and had previously been part of the UGA4 season, were selected.

### Hemagglutination-inhibition (HAI) assay

Blood samples were drawn from the participants on the day of vaccination (Day-0), preceding vaccine injection, and then again on the seventh and twenty-eighth days post-vaccination (Day-7 and Day-28). Hemagglutination inhibition (HAI) assays were conducted on the Day-0 and Day-28 blood samples against each of the vaccine strains as well as additional strains, as elaborated in a previous study (15). Briefly, Hemagglutination Inhibition (HAI) titer was ascertained by considering the reciprocal dilution of the final well consisting of non-agglutinated RBCs. For each plate, both positive and negative serum controls were incorporated. Using the World Health Organization (WHO) and the European Committee for Medicinal Products’ guidelines for evaluating influenza vaccines, seroprotected subjects were defined as an HAI titer equal to or greater than 1:40, and seroconverted subjects were defined as a fourfold increment in titer compared with the baseline, leading to a titer of 1:40 or more (42).

### Targeted bisulfite sequencing (TBS-seq)

DNA was isolated from each participant’s blood samples using the standard phenol-chloroform extraction approach previously described (43). For the construction of the TBS-seq library, 500 ng of the extracted DNA was used. The NEBNext Ultra II Library prep kit along with custom pre-methylated adapters (IDT) was used to perform adapter ligation and dA-tailing (9). The probes for targeted bisulfite sequencing (TBS-seq) were chosen based on specific parameters. The purified libraries were subsequently hybridized with the biotinylated probes under the given conditions: 2 min at 98°C; 14 cycles of (98°C for 20 sec; 60°C for 30 sec; 72°C for 30 sec); 72°C for 5 minutes; maintained at 4°C. Following the bisulfite treatment on captured DNA, PCR amplification was carried out using KAPA HiFi Uracil+. An examination of library quality was carried out using the high-sensitivity D1000 assay on a 2200 Agilent TapeStation. The libraries were then sequenced as 150 paired-end bases using two NovaSeq6000 (Sp lane).

A first set of probes was selected from previously published DNA methylation-based aging clocks (13). A second set was chosen to be within promoter regions of genes that respond to viral infections like SARS and influenza (44–47). A third set included immune cell type specific loci as established by the Blueprint Epigenome Project (48). The last set was chosen based on their correlation with metabolic phenotypes (49, 50). Their coordinates, based on GRCH38, can be found in the Supplementary Table 1.

### TBS-seq data process and methylation calling

Cutadapt (v2.10) was used to remove adapter sequences from the demultiplexed FastQ files (51). Subsequently, BSBolt (v1.3.0) was employed to align the reads to the GRCh38 reference genome (52). The markdup function in Samtools was used to eliminate PCR duplicates (53). The final step in this process involved calling methylation using the BSBolt CallMethylation function. To generate the methylation matrix, each CpG site was required to be covered by a minimum of 20 reads in all samples using the AggregateMatrix function in BSBolt.

### RNA sequencing and analysis (RNA-seq)

RNA was isolated from peripheral blood mononuclear cells (PBMC) samples. Libraries were prepared for samples that passed quality control using the KAPA stranded-mRNA kit. Prepared libraries were sequenced on the Illumina HiSeq3000 platform. After quality control by FastQC, reads were aligned to the GRCh38 human reference genome using STAR (54) and count tables were generated using R package Rsubread (55). For this analysis, transcripts were included with a minimum count of ≥ 5 reads in ≥ 10% of samples. To normalize sequencing depth and gene length, transcripts per million (TPM) was used to quantify gene counts. Differential gene expression analysis was performed using DESeq2 (56).

### Cell type deconvolution for PBMC

B cells, Granulocytes, Monocytes, Natural Killer cells, Naive T cells, and Non-Naive T cells, were selected as reference cell types within PBMCs. Their whole genome bisulfite sequences were obtained from a recently published DNA methylation atlas (17). Cell type abundance estimation was performed using CellFi, which employs non-negative least square methods for deconvolution (16). We first identified 37 differentially methylated regions that were specifically hypomethylated in each of the six cell types. We used non-negative least square regression to estimate cell type abundance using these 37 regions.

### Multivariate multiple regression model

A multivariate multiple regression model was used to account for different factors that affect DNA methylation and influenza vaccination response:

$$[M_{is}]=[F_{ic}]\times [\beta_{cs}]+\epsilon_{is}$$

Here, M is the methylation matrix, i is the ith sample, and s is the sth methylation site. F is the factor matrix, and c is the cth factor. For the factor matrix, we included both the demographic variables (age, gender and BMI) and the cell type compositions from the deconvolution method described above. To control for the year of the two cohorts, we also added a year variable where 0 is for UGA4 and 1 is for UGA5. The coefficients were estimated through least squares estimation, and is the random error term.


$${\hat{f}}_{ic}=m_{is}\times {\beta^{+}}_{cs}$$

For the prediction model, the pseudoinverse of the beta estimation was used (depicted as the plus sign) to predict the phenotype values based on the methylation levels. We utilized leave-one-out cross-validation to provide a more accurate prediction model assessment.

### Enrichment analysis of significant coefficients sites

We selected the methylation sites that have significant coefficients for each trait after false discovery rate correction for multiple testing for both positive and negative coefficients separately. Gene ontology biological process enrichment was performed by mapping the significant sites to their proximal genes using biomaRt and then measuring enrichment of the gene sets using Enrichr and GREAT (57, 58). We used the entire set of target regions as the background set for these analyses. Transcription factor binding, histone marks variants, and chromatin accessibility overlap analyses were performed using the Cistrome Data Browser (59). Genomic region sets that overlap with the query set were also computed using LOLA (60).

## Figures and Tables

**Table 1:** Top 9 enriched pathways of significant positive HAI coefficients of the multimodal model. Significant sites were mapped to their proximal genes, and gene ontology enrichment was conducted against the full background set.

Pathway	Overlap	P-value	Overlap Genes
**Regulation Of Defense Response To Virus (GO:0050688)**	10/16	8.83382819583528E-09	ILRUN;PPM1B;RNF216;DHX9;ELM0D2;C1QBP;PCBP2;TNFAIP3;IFNLR1;ATG5
**Negative Regulation Of Response To Biotic Stimulus (GO:0002832)**	7/15	2.37558081599061E-05	ILRUN;PPM1B;C1QBP;PCBP2;TNFAIP3;ATG12;ATG5
**Negative Regulation Of Type I Interferon Production (GO:0032480)**	7/17	6.3909478276655E-05	ILRUN;PYCARD;PPM1B;RNF125;RNF216;ATG12;ATG5
**Regulation Of Type I Interferon Production (GO:0032479)**	7/22	4.19269386834906E-04	ILRUN;RNF125;DHX33;RNF216;ATG12;PQBP1;ATG5
**Negative Regulation Of Defense Response To Virus (GO:0050687)**	5/11	4.5122299575726E-04	ILRUN;PPM1B;C1QBP;PCBP2;ATG5
**Negative Regulation Of Immune Response (GO:0050777)**	5/12	7.31430042128143E-04	PCBP2;TNFAIP3;FURIN;ATG12;ATG5
**Regulation Of Defense Response To Virus By Host (GO:0050691)**	7/24	7.57836072328408E-04	RNF216;DHX9;SIN3A;TNFAIP3;IFNLR1;EIF2AK4;PQBP1
**Negative Regulation Of Protein Localization To Nucleus (GO:1900181)**	3/4	0.0011758126952240700	ILRUN;SIN3A;SUFU
**Negative Regulation Of Signal Transduction By P53 Class Mediator (GO:1901797)**	3/4	0.0011758126952240700	PRKN;KDM1A;SNAI1

## Data Availability

All the raw sequence FastQ data and processed methylation CGmap data are uploaded to Gene Expression Omnibus (GEO) and are accessible through GEO series accession number GSE263782. Metadata is uploaded as additional file 4 and 5 for UGA4 and UGA5. The raw RNA count matrix is uploaded as additional file 6.

## Abbreviations

BMI: Body mass index
DNAm: DNA methylation
DMR: Differential methylation region
GEO: Gene Expression Omnibus
HAI: Hemagglutination inhibition assay
LOOCV: Leave one out cross validation
NGS: Next-generation sequencing
PMBC: Peripheral blood mononuclear cells
RBC: Red blood cells
TPM: Transcripts Per Million
TBS: Targeted bisulfite sequencing
UGA: University of Georgia cohorts
